# An ectopic renin‐secreting adrenal corticoadenoma in a child with malignant hypertension

**DOI:** 10.14814/phy2.12728

**Published:** 2016-03-20

**Authors:** Abraham M. Kaslow, Anne Riquier‐Brison, Janos Peti‐Peterdi, Nick Shillingford, Josephine HaDuong, Rajkumar Venkatramani, Christopher P. Gayer

**Affiliations:** ^1^ Children's Hospital Los AngelesLos AngelesCalifornia; ^2^ Keck School of Medicine of USCLos AngelesCalifornia; ^3^ Baylor College of MedicineHoustonTexas

**Keywords:** Adrenal mass, aldosterone, ectopic renin, malignant hypertension, pheochromocytoma

## Abstract

A previously healthy 7‐year‐old male presented with hypertensive emergency, hypokalemia, and elevated plasma renin activity and aldosterone levels. There was no evidence of virilization or cushingoid features. MRI of the abdomen revealed a large (5 × 5 × 3 cm) peripherally enhancing, heterogeneous mass arising from the left adrenal gland. The patient was treated for a suspected pheochromocytoma. However, his blood pressure was not responsive to alpha‐blockade. Blood pressure was controlled with a calcium channel blocker and an angiotensin‐converting enzyme (ACE) inhibitor. A complete surgical resection of the mass was performed. Postoperatively, his blood pressure normalized and he did not require antihypertensives. On pathological examination, the tumor tissue stained negative for chromogranin and positive for renin. The final diagnosis was renin‐secreting adrenal corticoadenoma, an extremely rare adrenal tumor not previously reported in a pediatric patient. Malignant hypertension due to a renin‐secreting tumor may need to be distinguished from a pheochromocytoma if alpha‐adrenergic blockade is ineffective.

## Introduction

The following case report describes an exceedingly rare tumor in an even more rare age demographic: an ectopic renin‐secreting adrenal adenoma in a 7‐year‐old male child presenting with hypertensive emergency. The rarity of the tumor and age of presentation served as compounding factors that made this case particularly difficult from a diagnostic and treatment standpoint. The family consented to this case report. The differential diagnosis of hypertensive emergency in a pediatric patient with an adrenal mass includes pheochromocytoma, adrenal cortical adenoma, and neuroblastoma, among others.

At present, there exists a fine line between ordering the appropriate test and shotgun testing. In this particular case, the correct tests were ordered. The interpretation of the results, however, was difficult because they revealed such a rare diagnosis: ectopic renin secretion.

This case report aims to not only describe a physiologically, medically, and surgically interesting and rare tumor, but also to place ectopic renin‐secreting tumors on the differential diagnosis when presenting with elevated plasma renin activity (PRA), low aldosterone, and negative catecholamines. The consequence of this outcome will be to effect rapid treatment of the patient with appropriate angiotensin‐converting enzyme (ACE) inhibition and surgical removal.

## Case Report

A previously healthy 7‐year‐old boy presented to an outside hospital with altered mental status and seizures. He had history of weight loss, intermittent blurry vision, polyuria, and polydipsia for 2 weeks. His initial blood pressure was 220/130 mmHg. Computed tomography scan of the brain was normal. His seizures were controlled with lorazepam. He was started on a calcium channel blocker, nicardipine drip, and transferred to Children's Hospital Los Angeles (CHLA) for further management and work‐up of the hypertension.

At CHLA, physical examination was significant for tachycardia (136 beats/min) and hypertension (166/96 mmHg). The patient was noted to have a waxing and waning mental status and a hyperdynamic precordium. He had a normal respiratory and abdominal examination. There was no evidence of virilization. An abdominal ultrasound revealed a mass at the superior pole of the left kidney, concerning for an adrenal mass. Serum electrolytes revealed marked hypokalemia (2.1 mEq/L) and normal sodium (138 mEq/L). TSH, T4, and cortisol levels were normal. Further tests of serum and urinary catecholamines and of the renin–angiotensin aldosterone system (RAAS) were obtained to rule out pheochromocytoma as summarized in Table 1. These and all subsequent laboratory values were drawn while the patient was positioned supine. Initial values were drawn within 4 h of starting the nicardapine drip.

**Table 1:** Laboratory results

Serum/urine marker	Value	Age‐matched normal range
Urinary catecholamine breakdown products		
HVA (mg/g creatinine)	11.5	3–15
VMA(mg/g creatinine)	14.3	3–9
Serum catecholamines		
Epi (pg/mL)	213	456
NoEpi (pg/mL)	1247	1252
Spot urine catecholamines		
Epi (*μ*g/g creatinine)	52	4–32
NoEpi (*μ*g/g creatinine)	317	20–108
24‐h urine catecholamine		
Epi (*μ*g/24 h)	3	1–7
NoEpi (*μ*g/24 h)	66	5–41
24‐h urine metanephrines (*μ*g/24 h)	60	11–139
24‐h urine normetanephrines (*μ*g/24 h)	269	31–398
Plasma renin activity (ng/mL per h)	132.4	0.25–5.82
Serum aldosterone (ng/dL)	71	<9

HVA, homovanillic acid; VMA, vanillylmandelic acid; Epi, epinephrine; NoEpi, norepinephrine.

## Initial Diagnosis

**Figure 1:**
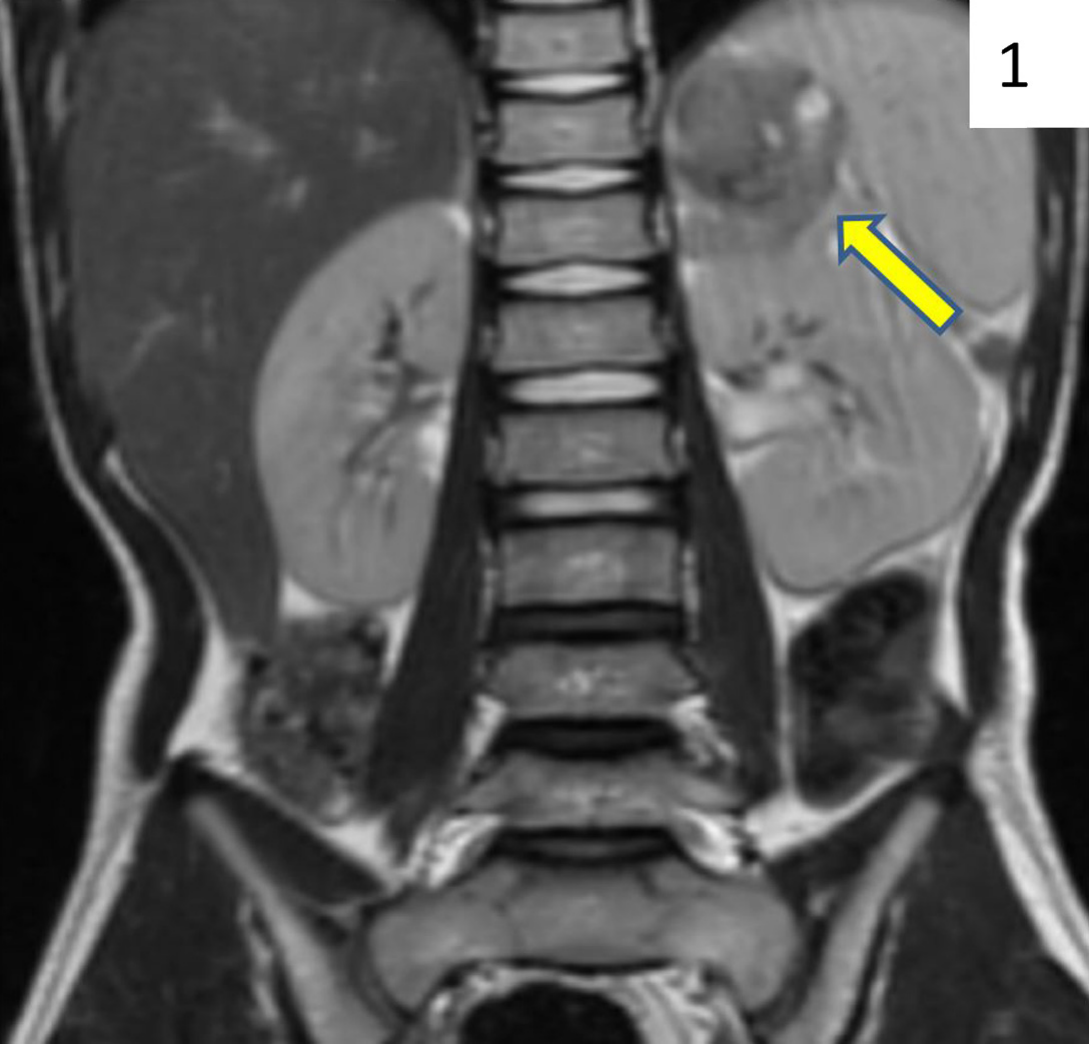
T2‐weighted MRI of abdomen, coronal view. The arrow indicates a 5‐cm lesion above the left kidney consistent with an adrenal mass.

Renin (PRA) and aldosterone levels were significantly elevated. However, markers of sympathetic output were within normal limits with the exception of VMA (vanillylmandelic acid) (Table 1). This unusual constellation of findings led us to the following potential differential diagnoses: pheochromocytoma, an extra‐ or intrarenal tumor exerting mass effect on the renal artery, or a primary renin‐secreting tumor. A renal ultrasound with Doppler demonstrated normal flow within the renal vessels. An MRI confirmed a 5 × 5 × 3 cm peripherally enhancing, heterogeneous adrenal mass with internal hemorrhage (Fig. 1). A metaiodobenzylguanidine (MIBG) scan, which is used to assess for neuroblastoma, was negative for any uptake outside the location of normal adrenal tissue and a CT scan of the chest was negative for metastatic disease.

Once the blood pressure was controlled, the patient was weaned off the nicardipine and started on the ACE inhibitor enalapril and calcium channel blocker amlodipine. Amlodipine was eventually transitioned to the alpha‐blocker phenoxybenzamine due to the elevated VMA level and the concern for pheochromocytoma. The elevated plasma renin activity was postulated to be due to ectopic renin secretion from the tumor, although pheochromocytoma remained highest on the differential. The patient was discharged to home on enalapril 2.5 mg PO BID ( by mouth two times daily )and phenoxybenzamine 2.5 mg PO TID (by mouth three times daily) for blood pressure control, as well as potassium supplementation for hypokalemia. Surgical resection was planned for after 2 weeks of alpha‐blockade.

The patient's blood pressure was not well controlled as an outpatient despite good medication compliance. He was admitted twice with headache and hypertension. The dose of phenoxybezamine was increased gradually to 40 mg PO TID and dose of enalapril increased to 7.5 mg PO BID. After controlling his blood pressure as a presurgical inpatient, the patient underwent an open left adrenalectomy with retroperitoneal lymph node sampling. Despite aggressive manipulation of the tumor during the dissection, there was no hypertensive response intraoperatively, nor were additional blood pressure medications required intraoperatively, both suggesting that this lesion was not a pheochromocytoma. The adrenal vein was not sampled intraoperatively.

## Outcome

Following surgery, his blood pressure medications were stopped. On postoperative day 2, plasma renin activity and aldosterone levels had normalized to 1.02 ng/mL per h and <1 ng/dL, respectively. Sodium (135 mEq/L) and potassium (3.9 mEq/L) levels also normalized. The patient was discharged home on postoperative day 4. There were no recurrent hypertensive events.

**Figure 2:**
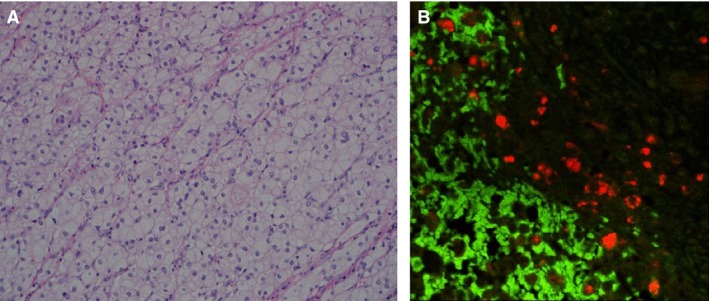
Histochemical analysis of adrenal mass. (A) H&E stain, 200× magnification. This section shows a monotonous population of neoplastic cells with abundant clear cytoplasm and small bland appearing nuclei. (B) 200×. Renin immunolabeling is demonstrated in red and is diffusely present in the tumor section. Normal adrenal tissue displays green autofluorescence.

Pathologic sections of the tumor demonstrated a well‐circumscribed, well‐vascularized mass with a fibrous capsule. Histologic examination of the tumor showed classic neoplastic characteristics, including mitotic figures and prominent nuclei. Immunohistochemical staining was positive for the calcium‐binding protein calretinin and the gonadal hormone inhibin, and negative for the neuroendocrine secretory protein chromogranin. Staining for the inflammation and tumor marker S100 did not demonstrate the classic sustentacular pattern expected in a pheochromocytoma (Lloyd 2011). This pattern of immunostaining did not fit the pattern of any standard diagnosis including pheochromocytoma. Sections of the tumor were subsequently stained for renin using a rabbit polyclonal antibody (Anaspec, Fremont, CA). It was diluted 1:100, and sections were incubated overnight at 4°C. The immunoreactivity was detected with a donkey anti‐goat secondary antibody conjugated to Alexa‐594 (red fluorophore, Invitrogen, Carlsbad, CA), diluted to 1:500, incubated at room temperature for 1 h. As shown in Figure 2, the tumor section exhibited several renin positive (red) cells containing large renin storage granules. These renin cells are not present in the control area of the adrenal tissue (green autofluorescence from endogenous NADPH). Based on these immunohistochemical results, coupled to the elevated plasma renin activity, the final pathologic diagnosis was an ectopic renin‐secreting adrenal corticoadenoma; ectopic because renin is normally secreted from the juxtaglomerular cells of the renal arterioles in the kidney, not the adrenal gland (Corvol et al. 1994). Lymph nodes were negative for disease.

## Discussion

This patient's presentation and laboratory values did not fit into any of the classic, albeit rare, adrenal tumors. The patient presented with malignant hypertension, hypokalemia, and an aldosterone‐to‐renin ratio (ARR) of 0.53 indicative of secondary hyperaldosteronism, a condition of excessive renin secretion unresponsive to normal negative feedback mechanisms. This is most commonly observed in juxtaglomerular cell tumors and renal artery stenosis, neither of which our patient exhibited. Catecholamines and prostaglandins act through physiologic mechanisms to stimulate aldosterone synthesis (Willenberg et al. 2008). The release of aldosterone by adrenocortical cells is known to be controlled in part by endothelial cell‐derived factors and adipokines. Such mechanisms aid in explaining previous literature, which has demonstrated that pheochromocytoma‐associated elevated catecholamine levels can cause an increase in plasma renin activity levels (Vetter et al. 1976; Plouin et al. 1988). Additionally, catecholamines lower extracellular potassium by activating cellular uptake (Youn and McDonough 2009). The patient, however, did not demonstrate catecholamine levels consistent with a pheochromocytoma (Table 1). Although his VMA was moderately elevated, a single plasma VMA measurement has not been shown to have diagnostic utility in the diagnosis of pheochromocytoma (Guller et al. 2006; Boyle et al. 2007).

Laboratory investigations can aid in narrowing the broad differential diagnosis of an adrenal mass with elevated blood pressures. In the absence of familial pheochromocytomas, 24‐h urine fractionated metanephrines is most specific for pheochromocytoma (Young 1993) and has a sensitivity of 98% and a specificity of 98% (Kudva et al. 2003). In this case, there was no family history and the patient's 24‐h urine metanephrine and normetanephrine values were normal. The combination of low clinical suspicion and normal laboratory values should have decreased the suspicion for a pheochromocytoma (Young 1993). If the diagnosis of pheochromocytoma is clinically suspected and catecholamine measurements are within the normal range, Guller et al. recommends MIBG scanning, which was negative in our patient (Guller et al. 2006). Persistently elevated PRA and aldosterone levels in the setting of uncontrolled hypertension and increasing alpha‐blockade point to an underlying angiotensin II‐mediated vasoconstriction. Additionally, while alpha blocked, this patient never developed overwhelming tachycardia during his hypertensive crises, indicating that there was no *β*1 activation. This is inconsistent with pheochromocytoma.

While it is known that the juxtaglomerular apparatus of the kidney secretes renin that promotes the systemic effects of angiotensin II, several organs, including the adrenal gland, brain, heart, and blood vessels, contain a regional coincidence of mRNA and protein for renin, angiotensinogen, ACE, and angiotensin receptors (Bader and Ganten 2008). The scientific significance of these renin–angiotensin systems (RAS) is that the local angiotensin II (AngII) produced at or near the target cell has specific local, rather than general, systemic actions (Campbell 1987, 2014). If cells in the adrenal gland produce renin, it can be postulated that if these renin‐producing cells undergo neoplastic change it could result in unregulated renin production. Renin‐producing cells in the adrenal gland have been localized to the zona glomerulosa in rat models (Mulrow 1989), but have yet to be localized to a specific zone in a human tumor. As the adrenal cortex does not typically secrete renin, this may be the product of pluripotent stem cells in this region. We were unable to determine the location within the adrenal cortex of the renin‐secreting cells. This unregulated extra‐renal renin production also could act systemically on plasma angiotensinogen leading to increased AngII‐mediated vasoconstriction and aldosterone‐mediated sodium reabsorption at the expense of elevated potassium excretion. This scenario may account for the constellation of symptoms and laboratory values our patient exhibited.

The increased plasma renin activity measured in this patient was associated with a high abundance of anti‐renin antibody immunoreactivity in the adrenal tissue (Fig. 2). The antibody used also detects the renin precursor prorenin. When the prorenin receptor is activated, the conversion of angiotensinogen to Ang I is increased fourfold (Nguyen et al. 2002). A recent study reported high levels of prorenin receptor in normal human adrenal cortex, and plasma prorenin was detected in hypertensive patients with primary aldosteronism (Recarti et al. 2015). Taken together, these findings illustrate the potential for adrenal‐produced renin and prorenin to contribute to the development and maintenance of hypertension.

An extrarenal renin‐secreting tumor in a child is rare. CHLA is a major pediatric referral hospital and has seen over 50 pediatric patients with pheochromocytoma since the late 1960s, all of which have arisen from the adrenal gland. During this time, there has been only one instance of a renin‐secreting tumor in a child, reported in 1979 (Warshaw et al. 1979). This patient was a 14‐year‐old girl with malignant hypertension. She had a renin‐producing juxtaglomerular cell tumor not involving the adrenal gland. She was treated with surgical nephrectomy and her hypertensive episodes, as well as renin levels, resolved.

A case report of a previously healthy 19‐year‐old male describes a similar scenario as our patient (Kawai et al. 1998). He presented with hypertensive emergency, elevated renin and aldosterone levels, and an adrenal mass. His blood pressure was controlled on an ACE inhibitor. After surgical removal, the tumor stained positive for renin, suggesting an ectopic source of renin secretion. Similar to our patient, he required no postoperative blood pressure medications. This case report lists 25 other instances of ectopic renin secretion from 1970 to 1994. Only three of these, including their patient, showed ectopic renin from an adrenal neoplasm, and none were in the pediatric population. The other two patients were a 27‐year‐old female with an adrenocortical carcinoma and a 23‐year‐old female with an adrenal paraganglioma.

From 1995 until present, only one other case report describing similar adrenal ectopic renin secretion was found (Yamanaka et al. 2000). This report described a 57‐year‐old woman with generalized weakness and thirst who was found to have a renin‐ and sex‐steroid‐secreting adrenalcortical carcinoma. In addition to malignant hypertension, she presented with virilization and new onset hyperglycemia, consistent with diabetes. Furthermore, her tumor was diagnosed via tissue sampling from a metastatic lesion in her neck. Our patient did not have other endocrine manifestations or distant disease. The patient we describe here is the youngest patient with an ectopic renin‐secreting adrenal mass described in the literature.

## Perspectives

If a similar scenario were to present itself, controlling the blood pressure with ACE inhibition may result in less hospital days and, more importantly, shield vital organs from dangerously high pressures that can result in irreversible end organ dysfunction. While renin‐secreting adrenal tumors are exceedingly rare, they should be included in initial broad differential diagnosis given applicable clinical situations and laboratory values and the appreciation that there are local RAS systems in adrenal as well as other extrarenal sites. In the setting of malignant hypertension, pheochromocytoma must be considered since adequate blood pressure control is necessary prior to surgical intervention. Intraoperative hypertensive crisis can be life threatening. However, failed blood pressure management with adequate alpha‐blockade should provoke the physician to question the more common diagnosis of pheochromocytoma and consider extrarenal renin‐secreting tumor.

## Conflict of Interest

None declared.
